# The impact of intergenerational support on social participation patterns of older adults in rural China

**DOI:** 10.3389/fpubh.2024.1392900

**Published:** 2024-06-03

**Authors:** Ping Wang, Xin Cheng

**Affiliations:** School of Management, Xi’an University of Science and Technology, Xi’an, Shaanxi, China

**Keywords:** intergenerational support, rural older adults, social participation patterns, China, latent class model

## Abstract

**Objectives:**

This study aimed to examine the association between different dimensions of bi-directional intergenerational support and the social participation patterns of rural older adults, while also exploring the heterogeneity of these older adults by gender and age.

**Methods:**

Based on longitudinal survey data from the ‘Well-Being of Older People in Anhui Province (WESAP)’ in 2018 and 2021, this study used latent class analysis to identify social participation patterns and used multinomial logistic regressions to explore the relationship between intergenerational support and social participation patterns among rural older adults.

**Results:**

First, the social participation patterns of rural older persons can be divided into four categories: leisure type (9%), work type (11%), housekeeping type (57.9%) and family labor type (22.1%). Second, there is heterogeneity in the relationship between intergenerational support and social participation patterns in older people, among them, receiving financial support decreased the likelihood of older adults being categorized as work type and family labor type by 14 and 7.7%, respectively, while providing financial support increased the likelihood of older adults belonging to the family labor type by 7.5%; receiving caring support increased the likelihood of older parents being categorized as leisure type by 6%, while providing caring support decreased the likelihood of older parents being categorized as leisure type by 10%; emotional support is related to all patterns of social participation among older adults. Third, the effects of intergenerational support vary across subgroups based on gender and age.

**Conclusion:**

Two-way intergenerational support was significantly associated with social participation among rural older adults, and there was significant heterogeneity in this association. Children and older persons should be encouraged to strengthen two-way intergenerational mobility to give full play to the positive impact of different dimensions of intergenerational support on the social participation of older persons in rural areas, ultimately enhancing the quality of life for rural older adults.

## 1 Introduction

The global trend of population aging is the most significant demographic change in the world today, and China is no exception. According to a report from the National Health Commission, China’s population aged 60 years and older reached 267 million by the end of 2021, and it is expected that by 2035, this number will exceed 400 million, accounting for more than 30% of the total population, entering a stage of severe aging (1). The 14th Five-Year Plan for the Development of the National Aging Program and the Elderly Service System clearly states that it encourages older adults to continue to play a role, supports them to in engaging in productive activities, advocates a positive view of aging throughout society, and guides them to actively participate in family, community and social development activities (2). Social participation, as a pillar of active aging, plays a crucial role in the health (3–5) and quality of life of older adults (6, 7) and has a positive impact on cognitive abilities (8), life satisfaction (9), and subjective well-being among older adults (10). It has been shown that social support factors have a significant impact on the level of social participation of older adults (11), particularly intergenerational support, which constitutes almost all informal support and is central to the social support network of older adults (12). As paramount family members of older adults, children have a profound impact on the later life of their parents through intergenerational exchanges, particularly in economically disadvantaged rural areas, where intergenerational support is the most critical resource that older adults rely on (13). However, the current research still inadequately addresses the association between intergenerational support and social participation patterns of older people in rural settings. This study aims to provide a comprehensive assessment of the social participation activities of rural older adults, taking their specific time use and allocation to work outside, self-employed paid labor, household chores, personal entertainment, exercise and social connection activities as the basis for classifying their social participation patterns to explore the relationship between two-way intergenerational support and social participation patterns of rural older persons in China. This study made several contributions to the literature. First, it expands the relevant research on social participation among rural older adults by overcoming the classification method of social participation patterns based solely on content or frequency. The article integrates the time data of older adults’ daily lives and utilizes their time data for social participation activities to categorize social participation patterns. Second, it delves into the relationship between two-way intergenerational support between children and older adults and social participation patterns among older adults in the rural context of China, demonstrating the significant role of two-way intergenerational support. Furthermore, gender and age differences in this relationship were examined to facilitate the formulation of targeted policies by government departments, aiming to leverage intergenerational support in enhancing the social participation of rural older adults.

## 2 Literature review

### 2.1 Research on time diaries

The use of time in daily activities is closely associated with health and quality of life. Retrospective 24-h time diary information is essential for providing a snapshot of older adults’ participation in activities throughout the day (14). Investigating the time utilization of older individuals serves as an effective tool for assessing their patterns of social participation. Detailed time data contribute to a deeper understanding of older adults’ daily lives and hold the potential to provide practical evidence on how to enhance their social participation. Previous research has comprehensively explored the time utilization patterns of older individuals, encompassing a wide range of aspects, such as motivations for time allocation, influencing factors, gender disparities, age-related characteristics, and cultural elements (15–17). However, investigations into the social participation patterns of older adults have predominantly focused on the content and frequency of their engagement in activities (18, 19), often overlooking the temporal aspects of older adults’ involvement in social participation endeavors.

### 2.2 Theoretical analysis of intergenerational support and social participation in older adults

Several theoretical frameworks, including social exchange theory and socioemotional selectivity theory, have been used to explain the relationship between intergenerational support and older persons’ social participation. Social exchange theory suggests that intergenerational support is a reciprocal interaction in which parents and children exchange resources based on their respective needs (20). The reciprocal relationship, where parents raise children and children support parents, encourages older adults to actively participate in society. For example, in return for their children’s support, older parents in rural China tend to provide their children with housework care or financial help, thereby increasing the likelihood of active social participation to maintain their advantageous position in social exchanges (21). This positive intergenerational exchange is essential for the psychological well-being and happiness of older people. Establishing close relationships and positive interactions with their children allows older individuals to have a more fulfilling mindset, facilitating increased positive emotions (22) and, consequently, a greater likelihood of social participation and improved life satisfaction. Socioemotional selectivity theory points out that people choose emotional experiences in social interactions to fulfill their emotional needs (23). This implies that individuals are more likely to engage with people or things that provide positive emotional experiences for emotional fulfillment (24). The theory emphasizes the importance of emotional fulfillment and support in the social interactions of older adults. As one of the closest relatives to older adults, children often provide substantial emotional support. Intergenerational interactions often provide substantial emotional support for older people, contributing to the enhancement of their emotional well-being, boosting their confidence (25), and motivating their participation in diverse activities.

### 2.3 Empirical study on the impact of intergenerational support on social participation in older adults

Previous studies have shown that access to financial resources, social–emotional interactions, and caregiving support are critical for older adults’ social participation (4). However, in actual studies, there were differences in the effects of different dimensions of intergenerational support on different social participation activities of older adults.

First, in terms of the impact of financial support. Studies have shown that receiving financial support from their children is significantly associated with greater social participation among older adults (26) and positively affects the frequency of their participation in family activities (27). Some scholars have argued that financial support provided by nonresident children and grandchildren considerably reduces the willingness of rural older adults to participate in labor (28), while whether children provide financial support to parents has no substantial effect on older adults’ care for grandchildren (29). Several studies, from the perspective of financial support provided by older adults, have shown that providing financial subsidies to children increases the likelihood of older adults participating in various productive activities (30) and noticeably increases the participation rate of urban older adults in economic activities (31). Second, from a caring support perspective, current research has focused on the effects of caregiving by older parents to their grandchildren on the participation in diverse activities. In the case of Chinese families, where most grandparents provide care for their grandchildren, this intergenerational caregiving will increase the chances of grandparents’ social participation (32), but it may also lead to role conflicts. The opposing viewpoint contends that if older adults are primarily responsible for caring for their grandchildren, this could crowd out older adults’ time and reduce their social activities (33), and concurrently lower the labor market participation rate of grandparents (34). In terms of access to caring support, access to child caring support could make older adults more dependent on their offspring, thus reducing their probability of social participation (35). However, some studies have suggested that household help provided by children to older adults positively promotes exercise activity among older adults (36). Third, from an emotional support perspective, intergenerational exchanges of emotions are positively correlated with older adults’ engagement in the care of their grandchildren (37). In addition, emotional tightness between children and parents positively promoted exercise behavior among urban older adults, and emotional support from offspring increased the likelihood of parental involvement in exercise activities to some extent (36).

### 2.4 Research review

In summary, the achievements of previous researchers have laid a robust foundation for this study, yet there are some notable limitations that warrant further investigation. First, while time data prove to be a valuable tool for comprehending the daily lives of older adults, there is a dearth of studies examining the characteristics of older adults’ social participation activities through the lens of time use. Second, numerous articles have solely delved into the impact of unidirectional intergenerational support on the individual social participation activities of older adults, overlooking the bidirectionality and mobility of intergenerational support. Furthermore, these studies have failed to integrate various social participation activities of older adults, neglecting the simultaneous and diverse nature of these activities. Last, previous research has focused limited attention on the heterogeneity of social participation among distinct groups of rural older adults, and comparisons to discern group differences are lacking. Therefore, this study focuses on the following questions: (1) How is time allocated to social participation activities among rural older adults, and what patterns of social participation are shaped by these allocations? (2) How do different dimensions of intergenerational support affect older adults’ social participation? (3) Are there gender and age differences in how intergenerational support is related to the social participation patterns of older adults?

## 3 Methods

### 3.1 Data sources

This study used mixed cross-sectional data from the ‘Well-Being of Older People in Anhui Province (WESAP)’ for two periods, in 2018 and 2021. The survey is conducted by the Population Research Institute of Xi’an Jiaotong University in the rural region of Chaohu city in Anhui Province. Its baseline survey was launched in 2011. During the field survey, investigators conducted household visits, inquiring about the personal circumstances of older individuals, and subsequently inquiring about the situation of each of their children in succession. The survey participants comprised rural older adults aged 60 years and above in the respective region. A stratified multi-level sampling method was employed, with samples drawn from townships and villages. Twelve townships were selected at random from a pool of 126 townships located within Chaohu city, 72 administrative villages were systematically sampled from the 12 townships, and within each administrative village, 15 older individuals aged 60–74 years and 10 individuals aged 75 years and older were chosen for participation. These samples will be followed up every 3 years. Following the research objectives, this study systematically excluded 46 participants without children, 91 participants with missing or abnormal data related to social participation activities, and 35 participants with missing or abnormal intergenerational support data. Additionally, 21 participants exhibited missing or abnormal values in other variables. Ultimately, a total of 2,591 valid samples were obtained.

### 3.2 Variable selection

#### 3.2.1 Dependent variables

The dependent variable, social participation is assessed by using time-use data through a retrospective 24-h diary method (14) to examine the lifestyle characteristics of older individuals in rural areas and understand their daily participation levels. This study argues that social participation should encompass all activities that benefit the development of older people themselves, their families and society. Therefore, the respondents were queried about their engagement in activities and the allocation of time from waking up to going to bed on the preceding day, with the exception of periods dedicated to rest and personal hygiene activities. The particulars of the selected activities are provided in Table 1.

**Table 1:** Distribution of specific categories and intensity indicators for each social participation activity.

Social participation activities	Specific categories of activities	Intensity of social participation activities (minutes)	Proportion (%)
Working outside	Leaving one’s place of residence to work in another place	Zero intensity: 0	87.07
		Low intensity: ≤480	6.45
		High intensity: >480	6.48
Self-employed paid labor	Own farm work, domestic animals, own business, hand production	Zero intensity:0	46.20
		Low intensity: ≤480	40.25
		High intensity: >480	13.55
Household chores	Grocery shopping and cooking, cleaning, washing and ironing, repairing, sewing, pets; care for grandchildren, spouse, children, parents, etc.	Zero intensity: 0	20.84
		Low intensity: ≤300	61. 60
		High intensity: >300	16.83
Personal entertainment	Read books, newspapers, watch TV, radio, Internet, etc.	Zero intensity: 0	24.35
		Low intensity: ≤240	57.20
		High intensity: >240	18.45
Exercise	Walking, square dancing, etc.	Zero intensity: 0	60.32
		Low intensity: ≤180	32.22
		High intensity: >180	7.45
Social connection	Interacting with people (talking on the phone, chatting, playing chess, playing cards, etc.); other social activities (village, town, meetings, gatherings, helping others, religious activities, etc.)	Zero intensity: 0	54.23
		Low intensity: ≤120	13.39
		High intensity: >120	32.38

The duration of activities in each category is measured in minutes. Due to the substantial variability and skewness in the distribution of time spent on various social participation activities, this paper re-encoded these continuous variables related to daily time use into three intensity levels within each category: zero intensity, low intensity, and high intensity, denoted as 1, 2, and 3, respectively. The criteria for selecting threshold points refer to the method proposed by Song Lu (38), which primarily considers mean values, medians, values insensitive to changes, or commonly accepted conventions. The specific distribution of participation intensity for each activity is detailed in Table 1. The classification of social participation types is obtained through latent class analysis based on zero intensity, low intensity, and high intensity levels for six categories of social participation activities, as described in the following sections.

#### 3.2.2 Independent variables

The independent variable in this study was bidirectional intergenerational support (39), which included financial support, caring support, and emotional support. The measurement of intergenerational support is described as follows:

1. Financial support received by older parents is the total monetary value of cash and in-kind provided by children to their parents in the 12 months prior to the survey. To avoid the effect of extreme values, the total economic value received by older parents is increased by “1,” and then the natural logarithm [ln(x + 1)] is taken as the financial support received by older parents in the model. The inverse is the financial support that older parents provided to their children.
2. Caring support for older parents encompasses assistance provided by children with household chores such as cleaning, washing clothes and dishes, as well as help with daily living such as bathing and dressing. The level of care was assessed in the questionnaire by asking about the frequency of assistance with housework and daily living provided by children. This study converted the four-level frequency of household and daily living assistance provided by children into specific counts: (1) done every day = 4; (2) at least once a week = 3; (3) several times a month = 2; (4) rarely = 1. The counts were separately accumulated for sons, daughters, daughters-in-law, and sons-in-law, resulting in scores for household and daily living assistance provided by children (the score ranges from 0 to 64 points). A higher score indicates a higher level of household and daily living assistance received by older adults.
3. Emotional support for older adults was measured using three questions: “In all respects, do you feel close to this child?” “In general, do you feel that you get along well with this child?” and “When you tell this child about your problems or difficulties, do you feel that he/she is willing to listen?.” Responses were assessed using a three-level scale (1 not close, 2 somewhat close, 3 very close; 1 not good, 2 okay, 3 very good; 1 reluctant, 2 sometimes willing, 3 willing), and scores for each question were summed to yield a total score (scores ranging from 3 to 9 points) that represented emotional connection with each child. The scores for all children were then summed to determine the level of intergenerational emotional communication.

#### 3.2.3 Control variables

Based on the literature, this study selected socio-demographic factors (40, 41), which include gender, age, education level, activities of daily living, household economic income, type of occupation, marital status, residence status, and number of children as control variables. The categorical variables included gender; age, indicating whether an individual is over 75 years old; educational level, denoting whether one has attended school or not; activities of daily living (ADL), reflecting the extent of difficulty the respondent faces in activities such as bathing, dressing and undressing, getting out of bed or getting up from a chair, walking around the room, going to the toilet, and eating. A code of 1 is assigned to indicate disability if there is difficulty or inability to perform any of these activities. Occupation type refers to whether the person is engaged in agricultural labor, marital status refers to whether the person has a spouse, and residency status refers to whether the person lives with another person. The others are continuous variables. Table 2 lists the specific definitions of these variables.

**Table 2:** Variable settings and descriptive statistics (*N* = 2,591).

Dependent variables	Average time spent (min)	Percentage (%)
Working outside	60.342	12.929
Self-employed paid labor	179.327	53.802
Household chores	169.429	79.159
Personal entertainment	147.430	75.646
Exercise	1.442	39.676
Social connection	113.515	45.773
**Independent variables**	**Average value**	**Standard deviation**
Receive financial support (thousand dollars)	7.474	2.654
Provide financial support (thousand dollars)	2.200	3.327
Receive caring support	1.751	4.618
Provide caring support	2.051	4.695
Emotional support	23.869	10.639
**Control variables**	**Average value**	**Standard deviation**
Gender (Female = 0; Male = 1)	0.513	0.500
Age (60–75 years = 0; over 75 years = 1)	0.256	0.437
Educational level (illiteracy = 0; educated = 1)	0.443	0.497
ADL (non-disability = 0; disability = 1)	0.096	0.294
Log of household economic income	7.534	3.581
Type of occupation (agricultural = 0; non-agricultural = 1)	0.250	0.433
Marital status (no spouse = 0; with spouse = 1)	0.774	0.418
Residence status (living alone = 0; living with others = 1)	0.821	0.384
Number of children	2.904	1.237

### 3.3 Empirical model

#### 3.3.1 Latent class model

Using latent class analysis to categorize patterns of social participation among older people. This method identifies latent variable values by analyzing individual values of manifest variables. As shown in Formula (1), the six manifest variables representing social participation activities for older adults include working outside, self-employed paid labor, household chores, personal entertainment, exercise, and maintaining social connection, denoted as A–F. The basic model is as follows:

(1) $$\pi_{\mathrm{ijklmn}}^{\mathrm{ABCDEF}}=\overset{T}{\underset{1}{\sum }}\pi_{t}^{X}\pi_{\mathrm{it}}^{\bar{A}X}\pi_{jt}^{\bar{B}X}\pi_{kt}^{\bar{C}X}\pi_{lt}^{\bar{D}X}\pi_{mt}^{\bar{E}X}\pi_{nt}^{\bar{F}X}$$

In the above equation, $\pi_{\mathrm{ijklmn}}^{\mathrm{ABCDEF}}$ represents the joint probability of the manifest variables, $\pi_{t}^{X}$ is the probability that the latent variable X is in class t, *t* = 1, 2, …T. $\pi_{\mathrm{it}}^{\bar{A}X}$ to $\pi_{nt}^{\bar{F}X}$ are the conditional probabilities of particular values of the indicator. Taking $\pi_{\mathrm{it}}^{\bar{A}X}$ as an example, $\pi_{\mathrm{it}}^{\bar{A}X}$ denotes the probability that an observer of potential class t has manifest variable A equal to i.

#### 3.3.2 Multinomial logistic regression

The results from the latent class model analysis indicate that the dependent variable in this study, social participation pattern, is an unordered categorical variable. Therefore, an unordered multinomial logistic regression model suitable for regression analysis with three or more categories is constructed. The basic model is shown in Formula (2):

(2) $$\ln\left(\frac{P_{j}}{P_{i}}\right)=\ln\left[\frac{P\left(y=j|x\right)}{P\left(y=i|x\right)}\right]=a_{j}+\overset{n}{\underset{k=1}{\sum }}\beta_{jk}x_{k},j\in \left(1,2,\ldots ,n\right)$$

Here, $x_{k}$ is the independent variable in the model, k is the number of independent variables; $a_{j}$ is a constant term of the model, $\beta_{jk}$ is the regression model coefficient value, $\frac{P\left(y=j|x\right)}{P\left(y=i|x\right)}$ is the ratio of event occurrences for the selection variable and the baseline variable, $P_{i}$ and $P_{j}$ denote the probability values of the baseline and selection variables, respectively.

## 4 Results

### 4.1 Descriptive statistics

In terms of social participation activities, engaging in self-employed paid labor was the activity with the longest participation time, averaging 179.327 min, for a participation rate of 53.802%. Among these six categories of activities, household chores had the highest participation rate of 79.159%, with an average time spent of 169.429 min. In contrast, exercise had the shortest average duration, only 1.442 min. In terms of intergenerational support, the average value of receiving financial support was much greater than the average value of providing financial support. However, older adults were more likely to provide life care for their children, with an average value of 2.051. In general, emotional exchanges between older parents in rural areas and their children score high. The descriptive statistics for the other variables are shown in Table 2.

### 4.2 Latent class analysis

To comprehensively analyze the diversity of older individuals’ social participation activities, this study estimated model parameters for latent class numbers ranging from 2 to 5. As shown in Table 3, when the number of classes is set at 4, the latent class model exhibits the lowest AIC and BIC values, and the highest entropy, and the values of LMR and BLRT are significant at the 0.001 level. Therefore, the selection of 4 classes represents the optimal choice for categorizing the types of social participation activities among older adults. Subsequently, this study named and characterized each class based on the conditional probability features of the latent classes. Table 4 presents the conditional probability distributions of older adults’ (non-zero intensity) social participation activities. Accordingly, the four categories of social participation activities are categorized as follows: leisure type, work type, housekeeping type and family labor type.

**Table 3:** Comparison of latent class analysis model fit.

Classes	AIC	BIC	ABIC	ENTROPY	LMR	BLRT
2	25761.454	25907.949	25828.517	0.502	0.0000	0.0000
3	25452.038	25674.711	25553.974	0.691	0.0000	0.0000
4	25217.134	25515.983	25353.942	0.750	0.0006	0.0000
5	25140.678	25515.705	25312.359	0.671	0.9378	0.0000

**Table 4:** Latent class probabilities and conditional probabilities of older individuals’ social participation.

Social participation activities	Category indicators	Leisure type	Work type	Housekeeping type	Family labor type
Working outside	Low intensity	0.009	0.408	0.028	0.025
	High intensity	0.026	0.592	0.001	0.000
Self-employed paid labor	Low intensity	0.110	0.309	0.465	0.449
	High intensity	0.097	0.004	0.000	0.551
Household chores	Low intensity	0.121	0.767	0.731	0.646
	High intensity	0.139	0.021	0.150	0.105
Personal entertainment	Low intensity	0.148	0.754	0.584	0.684
	High intensity	0.280	0.032	0.229	0.093
Exercise	Low intensity	0.068	0.213	0.490	0.228
	High intensity	0.123	0.000	0.050	0.010
Social connection	Low intensity	0.021	0.103	0.075	0.104
	High intensity	0.140	0.033	0.601	0.123
Latent class probabilities		0.090	0.110	0.579	0.221
Sample size		234	286	1,499	572

Category 1: Leisure type, accounting for only 9% of the sample. This group tends to be involved in individual leisure and interaction activities, as well as in domestic care to some extent. Category 2: Work type, with a share of 11%. Older individuals in this category exhibit significantly greater conditional probabilities of participating in work activities than other activity types. Category 3: Housekeeping type, with the highest percentage of 57.9%. Older individuals have a high conditional probability (0.731) of engaging in low-intensity household care activities. Category 4: Family Labor Type, comprising 22.1% of the sample. Older persons in this group spent most of their time engaged in paid family labor, while also participating in a greater number of household chores and personal recreational activities, alongside more involvement in household chores and personal leisure activities.

### 4.3 Regression results analysis

In this study, a multinomial logistic regression model is used to test the effects of different dimensions of intergenerational support on the social participation of rural Chinese older adults, and the estimation results of the model are shown in Table 5.

**Table 5:** Multinomial logistic regression analysis of social participation patterns of older adults (*N* = 2,591) (reference group: the housekeeping type).

Variables	Leisure type		Work type		Family labor type	
	RRR	S.E.	RRR	S.E.	RRR	S.E.
Receive financial support	1.001	0.041	0.860^***^	0.044	0.923^*^	0.038
Provide financial support	1.056	0.031	1.042	0.043	1.075^*^	0.033
Receive caring support	1.060**	0.027	0.876	0.064	0.953	0.034
Provide caring support	0.900**	0.047	1.075	0.071	1.019	0.052
Emotional support	1.068***	0.023	1.149^***^	0.053	1.058^*^	0.026
Control variables						
Gender (Female = 0)	1.508^***^	0.172	2.906^***^	0.471	0.918	1.314
Age (60–75 years old = 0)	1.114^***^	0.220	0.544^***^	0.183	0.682^*^	0.149
Education level (illiteracy = 0)	1.065	0.223	0.829	0.477	0.885	0.198
ADL (non-disability = 0)	2.449^***^	0.685	0.300^*^	0.142	3.932	0.688
Log of household economic income	1. 074^***^	0.020	1.420^***^	0.092	1.137^***^	0.030
Type of occupation (agriculture = 0)	0.775	0.240	2.689^**^	0.953	0.371^***^	0.131
Marital status (no spouse = 0)	0.619^*^	0.179	0.474	0.253	1.013	0.367
Residence status (living alone = 0)	1.515	0.451	1.909	1.065	2.365^**^	0.624
Number of children	0.662	0.125	0.300^**^	0.122	0.643^*^	0.139
Constant	0.203^***^	0.238	0.016^***^	0.026	0.460^***^	0.237
Loglikelihood	−2533.636					
R^2^	0.120					

*p < 0.1, **p < 0.05, ***p < 0.01, variable baseline category in parentheses.

First, the likelihood of belonging to the leisure type versus the housekeeping type was significantly different in terms of intergenerational bi-directional caring support and emotional support. Compared to that of the housekeeping type, for each point increase in the level of receiving caring support, the likelihood of older adults being the leisure type will increase by 6% (RRR = 1.060^**^). Conversely, for each point increase in caring provided to their children, the possibility of older adults being the leisure type will decrease by 10% (RRR = 0.900^**^). These older adults were more predisposed to maintain household activities and assume responsibility for the daily life of the family. For each point increase in the emotional support score, the probability of being a leisure type will increase by 6.8% (RRR = 1.068^**^). This group of older adults was more willing to engage in personal recreation and social interaction activities.

Second, the determining factors distinguishing work type from housekeeping one were receiving financial support and emotional support. Compared to that of the housekeeping type, for each unit increase in the natural logarithm of financial support received from children, the probability of older adults being the work type will decrease by 14% (RRR = 0.860^***^), while for each point increase in the emotional support score, the probability of older adults being the work type will increase by 14.9% (RRR = 1.149^***^).

Third, the significant factors determining the possibility of belonging to the family labor type versus the housekeeping type were all factors except for caring support. Regarding financial support, compared to the housekeeping type, for each unit increase in the natural logarithm of financial support received from children, the possibility of older adults being the family labor type will decrease by 7.7% (RRR = 0.923^**^); whereas for each unit increase in the natural logarithm of financial support provided to children, the likelihood of older adults being the family labor type will increase by 7.5% (RRR = 1.075^**^), indicating that this group of older adults is more prone to engage in paid work in their own homes. In terms of emotional support, for each one-point increase in the intergenerational emotional communication score, the probability of older adults becoming a family labor type will increase by 5.8% (RRR = 1.058^**^) vis-à-vis the housekeeping type.

### 4.4 Heterogeneity analysis

#### 4.4.1 Gender differences

Gender differences in the relationship between intergenerational support and social participation patterns of rural older adults were analyzed in Table 6 by dividing the sample into two subsamples of males and females based on gender. Compared to that of the housekeeping group, for each one-point increase in the score for receiving caring support and emotional support, the probability of female older adults belonging to the leisure type will increase by 9 and 6.1% (RRR = 1.090***, RRR = 1.061**), respectively, and for each one-unit increase in the natural logarithm of financial support provided to children, the likelihood of female older adults being the family labor type will increase by 11% (RRR = 1.110***). For male older adults, an increase of one unit in the natural logarithm of financial support from children decreased the likelihood of male older adults being the work type by 18.2% (RRR = 0.818**) compared to that of the housekeeping group. Similarly, for each point increase in the score of caring support received from children, the probability of older males being involved in work and family labor types will decrease by 23.5 and 13.7%, respectively (RRR = 0.765**, RRR = 0.863**). For each one-point increase in the score of caring support received from children, the likelihood of male older individuals becoming a leisure type will decrease by 19% (RRR = 0.810**), while an increase of one point in the emotional interaction score raised the likelihood of male older adults becoming a leisure, work, and family labor type by 9, 16.6, and 8.7% (RRR = 1.090**, RRR = 1.166***, RRR = 1.087**, respectively), compared to that of the housekeeping type.

**Table 6:** Results of gender heterogeneity analysis (reference group: housekeeping type).

Variable name	Female			Male		
	Leisure type	Work type	Family labor type	Leisure type	Work type	Family labor type
**RRR (S.E)**						
Receive financial support	0.989 (0.051)	0.853 (0.070)	0.953 (0.050)	0.979 (0.077)	0.818^**^ (0.072)	0.873 (0.070)
Provide financial support	1.032 (0.039)	1.092 (0.077)	1.110^***^ (0.043)	1.057 (0.058)	1.011 (0.064)	1.034 (0.060)
Receive caring support	1.090^***^ (0.035)	1.021 (0.107)	1.021 (0.046)	0.995 (0.032)	0.765^*^ (0.081)	0.863^**^ (0.049)
Provide caring support	0.921 (0.057)	0.884 (0.134)	1.021 (0.066)	0.810^**^ (0.079)	1.088 (0.105)	0.981 (0.089)
Emotional support	1.061^**^ (0.031)	1.253 (0.151)	1.048 (0.035)	1.090^**^ (0.038)	1.166^***^ (0.068)	1.087^**^ (0.045)
Control variables	Controlled					
Constant	0.252^***^ (0.179)	0.056^**^ (0.116)	0.537^***^ (0.042)	7.285^*^ (7.075)	1.181^**^ (1.511)	6.634^**^ (6.898)
Sample size	1,262			1,329		
R^2^	0.097			0.135		

^*^p < 0.1, ^**^p < 0.05, ^***^p < 0.01.

#### 4.4.2 Age difference

Table 7 shows the age differences in the relationship between intergenerational support and social participation patterns of older adults by dividing the sample into two subsamples based on age: the younger age group (60–75 years old) and the older age group (75 years old and older). In the younger age group of older adults, compared to the housekeeping type, each natural logarithm increase in receiving financial support will lead to a 13.7% (RRR = 0.863**) decrease in the likelihood of becoming the work type. However, for every one-point increase in the emotional communication score, the probability of the younger age group favoring the work type will increase by 13.7% (RRR = 1.137**). In the older age group of older adults, in comparison to those in the housekeeping type, each natural logarithm increase in providing financial support to children will result in an 8.2% (RRR = 1.082**) increase in the probability of leaning toward the family labor type. Moreover, each one-point increment in the score for receiving caring support will increase the probability of the older age group engaging in leisure activities by 6.8% (RRR = 1.068**), and each additional point in the emotional support score will increase the probability of them being the leisure type by 5.6% (RRR = 1.056**).

**Table 7:** Results of the age heterogeneity analysis (reference group: housekeeping type).

Variable name	60–75 years old			Over 75 years old		
	Leisure type	Work type	Family labor type	Leisure type	Work type	Family labor type
**RRR (S.E)**						
Receive financial support	0.996 (0.055)	0.863^**^ (0.051)	0.907 (0.046)	0.998 (0.057)	0.838 (0.085)	0.950 (0.067)
Provide financial support	1.064 (0.041)	1.023 (0.047)	1.063 (0.040)	1.019 (0.042)	1.099 (0.094)	1.082^**^ (0.055)
Receive caring support	1.048 (0.042)	0.878 (0.072)	0.944 (0.045)	1.068^**^ (0.032)	0.880 (0.119)	0.961 (0.051)
Provide caring support	0.862 (0.066)	0.987 (0.079)	0.969 (0.061)	0.920 (0.064)	1.252 (0.146)	1.094 (0.092)
Emotional support	1.039 (0.037)	1.137^**^ (0.064)	1.064 (0.037)	1.056^**^ (0.027)	1.077 (0.090)	1.011 (0.035)
Control variables	Controlled					
Constant	0.199^**^ (0.065)	0.232^***^ (0.234)	0.111^***^ (0.058)	0.114^***^ (0.074)	0.149^***^ (0.233)	0.727^***^ (0.536)
Sample size	1927			664		
R^2^	0.076			0.164		

^*^p < 0.1, ^**^p < 0.05, ^***^p < 0.01.

## 5 Discussion

### 5.1 Discussion of the results of the latent analysis

Rural older adults were classified into different types according to the marked differences in the time allocated to different social participation activities: leisure type, work type, housekeeping type and family labor type. Among them, more than half are characterized as the housekeeping type. Based on the observed time-use patterns, all four types of older adults are to some extent engaged in household chores or self-employed paid labor. This shows that the social participation of rural older persons is mainly focused on family activities. These rural older adults assume primary responsibility for daily household chores and contribute to the family’s well-being through their active engagement in these activities. Even within the leisure type, which represents the smallest largest proportion of participants, there is often an intertwining of domestic care activities. This pattern diverges significantly from Western cultural norms and aligns with the values typically associated with Chinese cultures, wherein the family unit holds great significance. In Chinese culture, the notions of family and kinship are highly esteemed (42). Consequently, rural older adults tend to prioritize family-oriented activities, deriving a heightened sense of self-satisfaction and overall well-being from their participation in such endeavors.

### 5.2 Discussion of the regression results

In terms of financial support, the more financial support older adults received from their children, the less likely they were to be the work and family labor type compared to the housekeeping type. This suggests that financial factors are essential to the social participation patterns of rural older adults, and directly impact their quality of life. Obviously, with more financial support from their children, older adults experience a decrease in their financial burden, subsequently leading to a reduction in their involvement in lower-level paid activities (43). This aligns with Maslow’s hierarchy of needs theory and effectively demonstrates the protective role of material capital in facilitating the social participation of older adults (44). However, it is noteworthy that as older adults provide more financial support to their children, their social participation patterns tend to lean toward the family labor type. This may be attributed to the strong sense of family values prevalent in rural areas, where older adults perceive providing financial support to their children as a family responsibility and an expression of love.

Caring support has a stronger explanatory power only for leisure and housekeeping types, and has no significant association with work and family labor types. Older adults who received a higher level of caring demonstrated a stronger inclination toward the leisure type than toward the housekeeping group. On the one hand, this could be attributed to the fact that when children help older adults with household chores and daily living tasks, they freed up their time to engage in social interactions and activities such as exercise. On the other hand, older adults who require assistance with daily living tasks often have poorer physical health (35) and can only participate in comfortable leisure activities. Moreover, older adults who provided more caring support were more likely to be of the housekeeping type. This can be explained by the fact that if older adults live with their children, they typically assume primary household responsibilities and may also need to care for grandchildren, which leads them to dedicate the majority of their time to family-related activities.

Emotional support is strongly correlated with various patterns of social participation among rural older adults. Older adults who experienced higher levels of emotional interaction with their children exhibited greater likelihoods of being engaged in leisure, work, and family labor types, as opposed to housekeeping one. Personal recreation and social interaction activities hold paramount importance for older adults in expanding their social networks. Due to the limited forms of emotional communication available to rural older individuals, intergenerational interaction serves as a primary source of psychological support for aging parents. Therefore, when children provide adequate encouragement (45), older adults are more willing to leave the home, concentrate on self-development, and actively partake in social activities. Emotional support represents a mutually comforting bond between older adults and their children, leading to psychological gratification as they share moments of joy and sorrow. Close emotional communication helps older adults cultivate a more enriched mindset, which contributes to the enhancement of their positive emotions (46). Consequently, this motivates their participation in work activities and paid labor within the family, enabling them to continue making contributions to both the family and society.

### 5.3 Discussion of heterogeneity

Regarding gender differences in the relationship between intergenerational support and older adults’ social participation patterns, this study argues that, for female older adults, they are traditionally expected to fulfill the roles of mother and wife within the family and society. When they receive more daily care, they have more leisure time available for recreational activities. As the economic income of rural female older adults is considerably lower than that of their male counterparts, they often need to engage in more work to bridge the gap in providing financial support to their children (47). On the other hand, for male older adults, who are expected to bear financial responsibility for their family, the relationship between receiving financial support and caring support and their participation in paid social activities is more pronounced. This can be attributed to the traditional Chinese cultural perspective on the gender-based division of labor and the distribution of family responsibilities among rural older adults (48, 49). Furthermore, the presence of rich emotional communication between older parents and their children facilitates their engagement in leisure and self-development activities. This may be because satisfying the emotional needs of rural older adults is a crucial element in enhancing their well-being in old age. However, emotional support only has a substantial effect on the participation of older male adults in economic activities. This could be because it is primarily male older adults who engage in economic participation, and their involvement in work often distances them from their relatives. A high level of bidirectional intergenerational emotional communication ensures that they receive better and more timely moral support and life protection from their children.

Regarding age differences, older adults in the younger age group who received higher levels of financial support were less likely to engage in outside work activities, whereas older adults in the older age group who provided more financial support tended to engage in more family labor. For older adults in the younger age group, the provision of greater economic support by their children satisfies their material needs, and younger older adults with good physical health often bear the responsibility of caring for grandchildren and managing household chores (25). These two factors contribute to their reduced likelihood of engaging in work outside the home. Older adults in the older age group, they continue to participate in paid home-based work to provide financial support to their children, which may be attributed to the interaction between older persons and their children, as well as traditional beliefs that providing material assistance to their children necessitates their engagement in paid home-based work for financial income. With respect to caring support, older adults in the older age group who received more caring support were more prone to participate in leisure and recreational activities. This may be because children taking care of their daily lives provide them with more companionship, which in turn increases older adults’ self-confidence in participating in society. Regarding emotional support, older adults in the younger age group with higher emotional communication scores were more likely to work type than were those in the housekeeping group, but more emotional support for older adults in the older age group significantly increased their likelihood of participating in leisure and recreational activities. For older adults in the younger age group in good health, frequent emotional communication with their children instills a stronger sense of self-worth, and motivation to participate in economic activities. As older adults transition from the younger to the older age group, their physical functioning declines. Therefore, having more emotional support can help them increase their self-confidence and overall well-being (22) and motivate them to engage in leisure and recreational activities.

### 5.4 Limitations

This study has several limitations. First, this study uses cross-sectional data, which makes it difficult to verify causal relationships. Second, the analysis sample is only from rural areas in Chaohu city, so the generalizability of this conclusion requires further investigation. Third, chronic diseases or mobility and functional limitations may affect the social participation patterns of older adults. However, these factors have not been adequately considered in the current study. Furthermore, due to the limitations of the research objectives, this study has not explored the mechanisms through which intergenerational support influences the social participation patterns of rural older adults.

## 6 Conclusion

First, the social participation patterns of rural older adults in China can be categorized as follows: leisure type, work type, housekeeping type and family labor type. Second, significant variations are observed in the effects of different dimensions of intergenerational support on various social participation patterns. These findings highlight the profound impact of bidirectional intergenerational dynamics within rural families on the social participation of older adults. Third, there are gender differences in the impact of intergenerational support on the social participation patterns of rural older adults. These differences are consistent with gender role socialization theory. Fourth, there are age-related disparities in the impact of intergenerational support on the social participation patterns of rural older adults. This divergent effect implies that as contemporary society evolves, the level of differentiation among rural older adult subgroups progressively intensifies, and the specific impact paths and consequences of intergenerational support on social participation patterns are differ.

Based on the above conclusions, this study proposes the following suggestions: (1) Relevant authorities should introduce more comprehensive and targeted social security policies covering the economic, caregiving, and emotional needs of older adults. Improving social security levels for rural older adults and encouraging those engaged in single-intensity activities to participate widely in various social activities are recommended. (2) Recognize the significant role of children’s support in the social participation of rural older adults. The legislation and institutional construction for family support system should be strengthened, and the obligations and rights of children should be acknowledged to provide support from a legal perspective. Complementary measures should encourage and support children in their positive roles in family care, such as implementing economic and holiday compensation policies. (3) Reasonably allocate resources for reciprocal intergenerational support, fostering a friendly family environment. Encourage intergenerational flow between children and older adults and leverage different intergenerational support combinations for positive effects on the social participation of older adults. (4) Understand the diverse needs of older adults and enhance the multidimensional nature of social participation. The different age groups of older adults suggest adapting support forms that meet their needs, respecting the autonomy of older adults in their choices. Considering gender differences, society should focus on the needs of older women, encouraging their diversified involvement in social activities.

## Data availability statement

The data analyzed in this study is subject to the following licenses/restrictions: the usage of data for this study requires permission from the Population Research Institute of Xi’an Jiaotong University. Requests to access these datasets should be directed to XC, 22202097036@stu.xust.edu.cn.

## Ethics statement

Ethical approval was not required for the studies involving humans because the use of non-identifiable secondary data in our study, formal approval from the Institutional Review Board is not required. The studies were conducted in accordance with the local legislation and institutional requirements. The participants provided their written informed consent to participate in this study.

## Author contributions

PW: Funding acquisition, Supervision, Writing – reviewing & editing. XC: Data curation, Formal analysis, Software, Writing – original draft.
